# Salinity can change the lipid composition of adult Chinese mitten crab after long-term salinity adaptation

**DOI:** 10.1371/journal.pone.0219260

**Published:** 2019-07-03

**Authors:** Xiaowen Long, Xugan Wu, Shaicheng Zhu, Haihui Ye, Yongxu Cheng, Chaoshu Zeng

**Affiliations:** 1 Centre for Research on Environmental Ecology and Fish Nutrition of Ministry of Agriculture and Rural Affairs, Shanghai Ocean University, Shanghai, China; 2 College of Ocean and Earth Sciences, Xiamen University, Xiamen, China; 3 Shanghai Engineering Research Center of Aquaculture, Shanghai Ocean University, Shanghai, China; 4 National Demonstration Centre for Experimental Fisheries Science Education, Shanghai Ocean University, Shanghai, China; 5 College of Science & Engineering, James Cook University, Townsville, Queensland, Australia; Zhejiang University College of Life Sciences, CHINA

## Abstract

The Chinese mitten crab (*Eriocheir sinensis*) is an euryhaline crustacean, whose adults migrate downstream to estuaries for reproduction. Lipids are believed to be involved in salinity adaptation during migration. This study investigated the effects of different salinities (0, 6, 12, and 18‰) on the total lipids, neutral lipids, and polar lipids contents, and fatty acid profiles in the gonads, hepatopancreas, and muscles of adult *E*. *sinensis* after 40 days of salinity adaptation. The results showed that the males and females from 12‰ treatment had the highest contents of total lipids and neutral lipids in their hepatopancreas and total lipids in the muscles. Notably, salinity had a greater effect on the fatty acid profiles in the hepatopancreas compared to that in the gonads and muscles. The male hepatopancreas treated with 18‰ salinity had the highest percentage of total n-6 polyunsaturated fatty acid (∑n-6PUFA) in both neutral lipids and polar lipids, while the percentage of total n-3 polyunsaturated fatty acid (∑n-3PUFA) in neutral lipids and polar lipids decreased significantly with increasing salinity in males. In females, the 0‰ treatment had the highest percentages of total saturated fatty acids in neutral lipids and polar lipids in the hepatopancreas, while the highest ∑n-3PUFA and ∑n-6PUFA in neutral lipids and polar lipids were detected in the 12‰ treatment group. In conclusion, brackish water could promote the accumulations of total lipids and neutral lipids in the hepatopancreas and change the fatty acid profiles of adult *E*. *sinensis*, particularly in the hepatopancreas after long-term salinity adaptation.

## Introduction

The Chinese mitten crab (*Eriocheir sinensis*) is an euryhaline crustacean that is mainly distributed along the Eastern coast of Asia to the Korean Peninsula [1, 2]. In addition, the crab has spread to the coastal ecosystems of Europe and America; thus, it is considered an invasive species [3, 4]. Under natural conditions, the juvenile *E*. *sinensis* inhabit fresh water environment, where they complete puberty molt from August to September. Then, the adult crabs after the puberty molt generally start the reproduction migration from the end of September to early October, and spend 1–3 months to reach the estuaries for reproduction. After completed copulation, the female crabs carrying the fertilized eggs gradually migrate further downstream for spawning and hatching in full marine environment. Subsequently, the hatched larvae develop through five zoeal stages to become megalops, and then the megalops develop into juvenile crabs and migrate upstream towards freshwater ecosystems, such as rivers and lakes [5, 6]. During the reproduction migration, the salinity increases from freshwater to brackish conditions; with the salinity in estuaries fluctuating substantially, as affected by tides and freshwater runoff from the rivers. For example, salinity of the Yangtze estuary reportedly ranges from 3.4‰ to 17‰ [7, 8], hence the osmoregulation and physiological status of *E*. *sinensis* will be impacted by the fluctuant salinity [9, 10]. Thus, the whole life history of the *E*. *sinensis* is modulated by fluctuating water salinity, making *E*. *sinensis* an ideal model organism to study the salinity adaptation mechanism in crustaceans.

Fluctuating salinity directly affects the osmolality of aquatic animals, which results in the metabolic changes. Energetic reorganization is one of the strategies used by aquatic animals to cope with environmental salinity changes [10–12]. Lipids are important nutrients in aquatic animals, which play important roles during salinity adaptation, being a primary energy source [13, 14] and structural components of membrane, determining membrane fluidity and its permeability regulation [15–17]. In addition, lipids are also the precursors for the biosynthesis of steroid hormones that regulate the reproductive endocrine system in aquatic animals [18–20]. Therefore, the adaptation of aquatic animals to different salinities may be understood by measuring the lipid compositions of the organs such as the gills, hepatopancreas, and muscle [21, 22]. Previous studies have investigated the effects of short-term salinity acclimation or abrupt salinity changes on the lipid composition of *E*. *sinensis*, and mainly focused on the lipid composition and phospholipid metabolism in the gills [23–26]. Recent studies reported the effects of long-term salinity adaptation on the fatty acid composition in the gills of adult *E*. *sinensis* [9, 10]; however, it is unclear whether salinity could affect the lipid compositions of other tissues after long-term salinity adaptation.

Previous studies have shown that the gonads of adult *E*. *sinensis* post puberty molt develop rapidly, and there are marked differences in the reproductive system and gonadal development pattern between male and female crabs [27, 28]. During gonadal development, the nutrients in the hepatopancreas of *E*. *sinensis*, especially lipids, are transported to the developing ovaries; however, this does not happen in male crabs [29, 30]. In addition, brackish water (6–18‰) could promote the gonadal development of *E*. *sinensis* during long-term salinity adaptation [9, 10]. It is likely that there might be gender differences in the lipid compositions of tissues after long-term salinity adaptation, and therefore, the effects of salinity on males and female crabs should be studied separately. According to the above summary, adult *E*. *sinensis* post puberty molt in early October were collected and subjected to four salinities (0, 6, 12 and 18‰) for forty days, to investigate the effects of long-term salinity adaptation on the total lipids, neutral lipids, polar lipids, and fatty acid profiles in the gonads, hepatopancreas, and muscle of adult *E*. *sinensis*. These results are envisaged to provide some useful information to understand the long-term salinity adaptation mechanism of *E*. *sinensis* during reproduction migration, and shed new lights on salinity adaptation mechanism for the studies in other euryhaline crustaceans.

## Materials and methods

### Experimental design

The adult *E*. *sinensis* post puberty molt was purchased from a crab farm in Yangchenghu Lake, Jiangsu province, China in early October. The adult crabs that completed puberty molt were determined based on the external characteristics: 1) > 70% area of the chelipeds is covered with hairs for adult male crabs; and 2) the petasma of adult males become hard and raised compared to immature males. For the females, the mature crabs were characterized by a yellowish green color on the carapace, and an abdominal flap that was semicircular in shape that was covered by short hair; in contrast, immature females had a khaki colored carapace, and the abdomen was more triangular [31]. The initial body weight of males and females ranged from 150 to 160 g and 100 to 120 g, respectively. The crabs were transported to Fengxian aquaculture research center, Shanghai Fisheries Research Institute, Shanghai, China. Two hundred and forty female and 240 male active and appendage-intact crabs were selected for experiment. Four salinity treatments, i.e. 0, 6, 12, and 18‰, were set up, and each treatment had two replicate tanks with 30 males or females stocked in each tank. Males and females were cultured separately, because in brackish water they could mate and cause spawning for female crabs [32], which may affect the normal physiological status of the crabs. The experiment was conducted in 16 indoor polyethylene tanks (length × width × depth = 2.5 m × 3.75 m × 1.0 m). Approximately 40% of the bottom area of the tanks was covered with 10–20 cm fine sand and pieces of polyvinyl chloride (PVC) tubes (diameter: 15 cm) were provided as shelters for the crabs. During the experiment, the water depth of each tank was maintained at 70 cm. The initial salinity in all tanks was 0‰, and following stocking of the crabs, the salinity in the tanks allocated for higher salinity treatments was gradually increased to the designated levels, at a rate of 3‰ day ^-1^ by adding brine.

During the experiment, all the rearing tanks were provided with continuous aeration and maintained under a photoperiod cycle of 12 h light: 12 h dark. Fluorescent lamps (40 W) were used as the light source. The crabs were fed daily at 18:00 with trash fish and the food residue was removed the next morning. The feeding amount was adjusted according to the water temperature and food residues. The feeding amount was around 3–5% of the total biomass when the water temperature was above 20°C, while the feeding amount was approximately 1–3% of total biomass when the temperature was between 15 and 20°C. The water temperature in each tank was measured daily at 12:00 and 22:00, and ammonia-N, nitrite, dissolved oxygen (DO), and pH were measured every 3 days. The water in each tank was exchanged based on the water quality. During the experiment, water quality parameters were maintained as follows: ammonia-N < 0.5 mg L^-1^; nitrite < 0.15 mg L^-1^; DO > 4 mg L^-1^ and pH 7.0–8.5.

### Sample collection

At day 40 of the experiment, the crabs were fasted for 24 h before sampling. Four crabs were randomly sampled from each tank; therefore, eight females and eight males were sampled from each salinity treatment. The body weights of crabs were measured using a digital balance (precision = 0.01 g). Subsequently, all the crabs were treated with the cold shock method to minimize suffering. The gonads and hepatopancreas of each crab were dissected out and weighted. Meanwhile, the muscle of each crab was carefully picked by hand. All the samples were stored at −40°C for later analysis.

### Total lipid extraction, separation, and fatty acid analysis

The gonads, hepatopancreas, and muscle samples of each crab was freeze-dried and pulverized. The total lipid content in the muscle and male gonads was relatively limited; therefore, to meet the requirements of total lipid content and fatty acid composition analysis, the muscle sample and male gonads of two crabs from the same tank were randomly pooled into one sample, respectively. Total lipids in the crab samples were extracted with chloroform-methanol (2:1, v/v) according to the method of Folch et al. (1957) [33]. Neutral lipids and polar lipids in the total lipids were separated by the solvent method using petroleum ether as the basal phase and 95% methanol as the recovery phase [29, 34].

The method of Shen (1992) [35] was used for the esterification of the above collected neutral and polar lipids samples. Briefly, 30–100 mg of the neutral lipids or polar lipids was solved with 2 mL petroleum ether: benzene (1:1, v/v), and an equal volume of 0.4 mol L^-1^ KOH-methanol solution was added, mixed, and left to stand for 10 min. Then, 10 mL distilled water was added, mixed, and left to stand for 30 min. The supernatant was then collected for fatty acid methyl esters (FAMEs) analysis. The FAMEs were analyzed on an Agilent 7890B-5977A gas chromatograph-mass spectrometer (GC-MS) with an Omegawax 320 fused silica capillary column (30 m × 0.32 mm ID × 0.25 um; Supelco, Billefonte, PA, USA). Helium was used as the carrier gas with a flow rate of 1.0 mL min^-1^. Aliquots (1.0 μL) were injected and the split ratio of the injector was 1:30. The temperature of the injector was kept at 240°C, while the column temperature was initially held at 40°C. It was then increased, at 10°C min^-1^, to 170°C and held for 1 min, followed by an increase at 2°C min^-1^ to 220°C and held for 1 min. It was then further increased at 3°C min^-1^ to the final temperature of 230°C and held for 5 min until all FAMEs had been eluted. The temperature of transfer line was maintained at 245°C. The ion-trap mass spectrometer was operated in electron impact (EI) mode and full scan monitoring mode (m/z 30–450). The MS source temperature was set at 230°C and the electron energy was set at 70 eV. The peaks were identified by comparing their retention times with known standards (Sigma-Aldrich Co., St. Louis, MO, USA). The fatty acid profile was expressed as the percentage of each fatty acid to the total fatty acids (% total fatty acids) based on the area percentage.

### Statistical analysis

Data are presented as mean ± standard error (SE). Homogeneity of variance was tested using Levene’s test. When necessary, arcsine-square root or logarithmic transformation was performed before analysis. Statistical analyses were conducted using ANOVA and Duncan’s multiple range tests were used as the means separation procedure in this study. *P* < 0.05 was regarded as statistically significant for any two treatments while Bonferroni correction was used to correct the *P* value of multiple tests for statistical significance [36]. All statistical analyses were performed using the SPSS statistics package software (version 16.0). Principal component analysis (PCA) was performed on fatty acid data using a statistical analysis module in the MetaboAnalyst 4.0 online software, which is freely available at http://metaboanalyst.ca.

## Results

### The contents of total lipids, neutral lipids, and polar lipids

There were no significant differences in the contents of total lipids, neutral lipids, and polar lipids, as well as the neutral lipids-polar lipids ratio (NL:PL), in the gonads of males among all treatments (*P* > 0.05, Table 1). In addition, no significant differences were found for the total lipids and neutral lipids contents and the NL:PL in the ovaries of females among all treatments (*P* > 0.05), while the polar lipids contents in the ovaries showed an overall decreasing trend with increasing salinity (*P* < 0.05). The highest contents of total lipids and neutral lipids in the hepatopancreas of male crabs were detected in the 12‰ treatment group (*P* < 0.05), while no significant differences were found for the contents of polar lipids and NL:PL among all treatments (*P* > 0.05). The total lipids, neutral lipids, and NL:PL in the hepatopancreas of females showed a pattern of ‘low-high-low’, with the highest levels detected in the 12‰ treatment group, while the highest and the lowest polar lipids contents were detected in the 0 ‰ and 12‰ treatment groups, respectively (*P* < 0.05). The male crabs from the 12‰ treatment group had the highest contents of total lipids and polar lipids in the muscles (*P* < 0.05), while no significant differences were found for the neutral lipids and NL:PL (*P* > 0.05). The neutral lipid contents and NL:PL in the muscles of females showed a pattern of ‘high-low-high’, with the highest and the lowest levels detected in the 0‰ and 12‰ treatment groups, respectively (*P* < 0.05).

**Table 1:** The contents of total lipids, neutral lipids and polar lipids (% wet tissue) and ratio of neutral lipids and polar lipids in the tissues of adult *E*. *sinensis*.

Items	Males				Females			
	0 ‰	6 ‰	12 ‰	18 ‰	0 ‰	6 ‰	12 ‰	18 ‰
Gonads								
Total lipids	1.083 ± 0.117	1.075 ± 0.185	0.857 ± 0.115	0.882 ± 0.038	15.561 ± 0.311	14.62 ± 0.206	15.68 ± 0.695	14.56 ± 0.127
Neutral lipids	0.055 ± 0.007	0.057 ± 0.013	0.034 ± 0.003	0.035 ± 0.010	8.117 ± 0.585	7.503 ± 0.554	8.415 ± 0.213	8.332 ± 0.224
Polar lipids	1.031 ± 0.110	1.018 ± 0.172	0.824 ± 0.111	0.860 ± 0.035	7.444 ± 0.165^a^	7.116 ± 0.786^ab^	7.261 ± 0.482^ab^	6.228 ± 0.031^b^
NL:PL	0.054 ± 0.008	0.055 ± 0.002	0.041 ± 0.003	0.041 ± 0.004	1.103 ± 0.097	1.080 ± 0.198	1.176 ± 0.052	1.340 ± 0.045
Hepatopancreas								
Total lipids	31.927 ± 0.262^B^	37.942 ± 2.141^AB^	40.822 ± 0.188^A^	36.637 ± 0.253^AB^	30.445 ± 1.700^b^	39.323 ± 1.622^a^	42.220 ± 2.19^a^	39.558 ± 1.988^a^
Neutral lipids	29.907 ± 1.465^B^	36.136 ± 2.257^AB^	38.646 ± 2.461^A^	34.694 ± 1.295^AB^	28.306 ± 1.965^b^	37.83 ± 1.438^a^	41.086 ± 1.995^a^	38.334 ± 1.346^a^
Polar lipids	2.020 ± 0.147	1.774 ± 0.126	2.004 ± 0.134	1.930 ± 0.133	2.013 ± 0.208^a^	1.410 ± 0.154^b^	1.080 ± 0.125^b^	1.170 ± 0.063^b^
NL:PL	15.604 ± 1.118	19.199 ± 1.254	17.978 ± 1.173	16.298 ± 1.027	14.241 ± 0.955^c^	29.270 ± 3.212^b^	40.580 ± 1.721^a^	33.121 ± 1.656^b^
Muscles								
Total lipids	0.926 ± 0.014^B^	0.966 ± 0.002^AB^	1.053 ± 0.061^A^	1.012 ± 0.048^AB^	1.051 ± 0.003	1.075 ± 0.034	1.101 ± 0.041	1.122 ± 0.064
Neutral lipids	0.027 ± 0.006	0.030 ± 0.005	0.027 ± 0.002	0.024 ± 0.002	0.029 ± 0.001^a^	0.024 ± 0.001^ab^	0.015 ± 0.002^c^	0.020 ± 0.001^bc^
Polar lipids	0.870 ± 0.013^A^	0.935 ± 0.020^AB^	1.028 ± 0.018^A^	0.989 ± 0.025^A^	1.024 ± 0.025	1.051 ± 0.014	1.085 ± 0.019	1.100 ± 0.028
NL:PL	0.031 ± 0.006	0.032 ± 0.005	0.026 ± 0.003	0.024 ± 0.002	0.028 ± 0.001^a^	0.023 ± 0.001^b^	0.014 ± 0.001^c^	0.018 ± 0.001^bc^

Data are presented as mean ± SE (n = 2). Within the male and female groups, values within the same row with different supesrcipt letters (capital letter for males and lower case letter for females) indicate significant difference; NL: neutral lipids; PL: polar lipids ratio.

### Fatty acid profiles of the gonads

**Fig 1:**
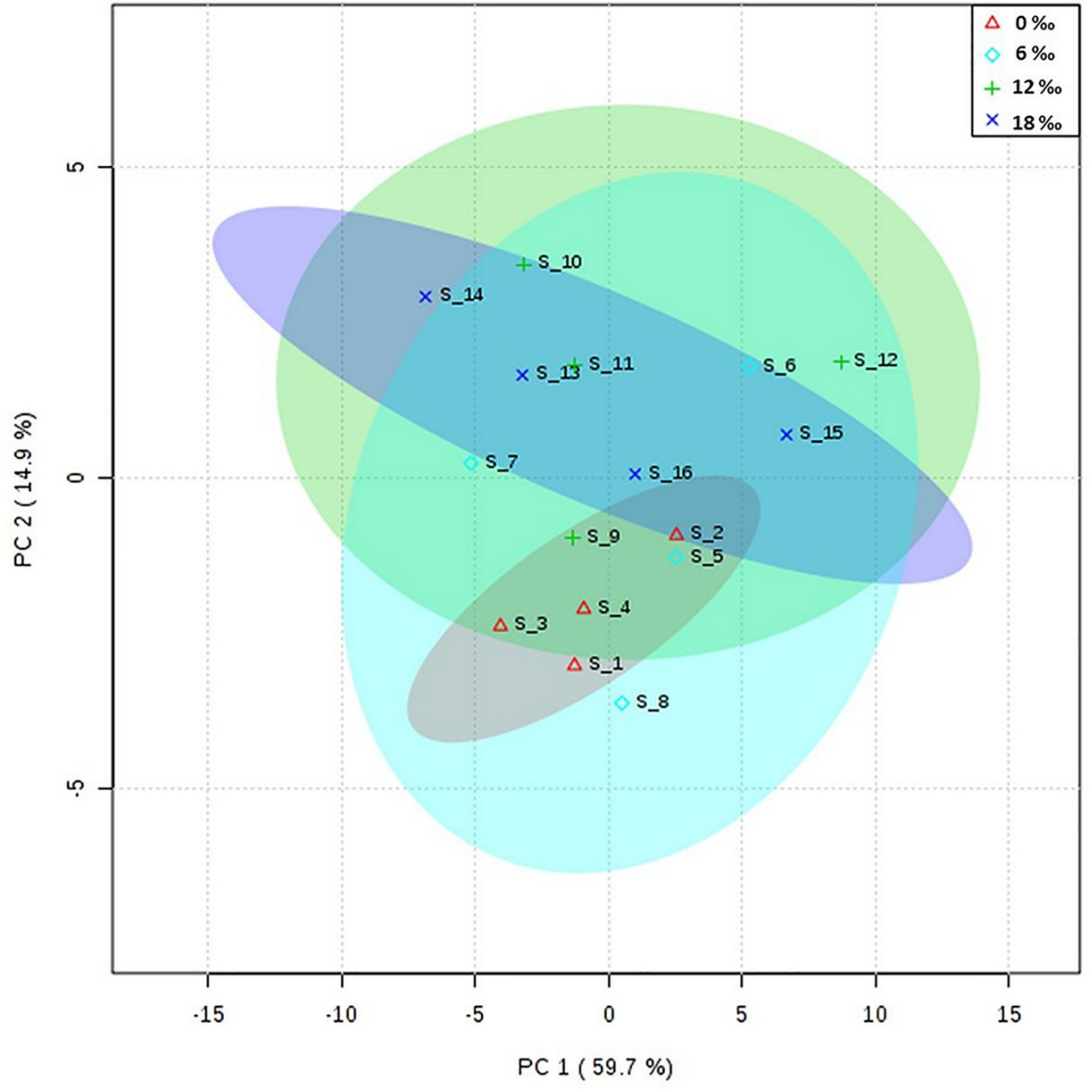
Principal component analysis (PCA) scores plot of fatty acids profiles in the gonads of adult male *E*. *sinensis*.

Principal component analysis (PCA) showed that the fatty acid profiles in the gonads of male crabs from the four salinity treatments were similar (Fig 1). For the fatty acid composition in the neutral lipids, there were no significant differences in the percentages of total saturated fatty acids (∑SFA) and total monounsaturated fatty acids (∑MUFA) in the male gonads among all treatments (*P* > 0.05, Table 2), while the highest percentages of C20: 5n3 (EPA) and total n-3 polyunsaturated fatty acid (∑n-3 PUFA) were detected in the 0‰ treatment group (*P* < 0.05). For the polar lipids, C18:0 showed a pattern of ‘high-low-high’, with the highest and the lowest levels being detected in the 18‰ and 6‰ treatment groups, respectively (Table 2).

**Table 2:** The principal fatty acid profiles (% total fatty acids) of the neutral lipids and polar lipids in the gonads of adult male *E*. *sinensis*.

Fatty acids	Neutral lipids				Polar lipids			
	0 ‰	6 ‰	12 ‰	18 ‰	0 ‰	6 ‰	12 ‰	18 ‰
C16:0	13.17 ± 0.28	13.22 ± 1.07	14.55 ± 0.30	13.77 ± 0.68	9.51 ± 0.06	10.34 ± 0.95	10.41 ± 0.87	9.00 ± 1.14
C18:0	8.17 ± 0.20	8.04 ± 0.14	7.97 ± 0.14	8.37 ± 0.13	7.37 ± 0.34^ab^	6.87 ± 0.36^b^	7.06 ± 0.17^b^	8.22 ± 0.22^a^
∑SFA	22.96 ± 0.49	22.91 ± 1.27	24.34 ± 0.22	23.85 ± 0.82	19.55 ± 0.27	19.91 ± 0.24	20.75 ± 0.66	20.32 ± 0.72
C16:1	3.76 ± 0.08	4.13 ± 0.58	3.78 ± 0.11	4.02 ± 0.47	3.44 ± 0.27	3.92 ± 0.76	3.73 ± 0.91	2.83 ± 0.82
C18:1n9	21.59 ± 0.10	20.98 ± 1.09	21.89 ± 0.31	22.04 ± 0.35	16.05 ± 0.54	17.06 ± 1.23	16.91 ± 0.71	16.76 ± 1.16
C18:1n7	5.24 ± 0.13	5.10 ± 0.25	5.44 ± 0.11	5.42 ± 0.39	3.72 ± 0.02	3.72 ± 0.15	3.74 ± 0.09	3.86 ± 0.37
C20:1n9	1.03 ± 0.01	1.13 ± 0.09	0.79 ± 0.04	0.89 ± 0.12	1.55 ± 0.05	1.26 ± 0.09	1.40 ± 0.18	1.40 ± 0.05
∑MUFA	32.25 ± 0.06	31.94 ± 1.86	32.60 ± 0.34	33.00 ± 1.12	25.74 ± 0.79	26.98 ± 2.05	26.95 ± 1.37	25.81 ± 2.33
C18:2n6	7.56 ± 0.30	7.26 ± 0.79	7.93 ± 0.29	8.79 ± 0.74	7.25 ± 0.64	7.45 ± 0.78	6.58 ± 0.32	7.03 ± 0.95
C18:3n3	0.83 ± 0.02	0.98 ± 0.05	0.89 ± 0.04	1.00 ± 0.05	0.77 ± 0.06	0.85 ± 0.12	0.72 ± 0.06	0.75 ± 0.08
C20:2n6	1.48 ± 0.02	1.47 ± 0.19	1.38 ± 0.02	1.52 ± 0.11	3.03 ± 0.01	2.79 ± 0.29	3.04 ± 0.08	3.03 ± 0.21
C20:4n6	11.47 ± 0.17	12.52 ± 0.98	11.57 ± 0.45	11.62 ± 1.74	13.54 ± 0.63	13.82 ± 1.43	14.39 ± 1.32	16.10 ± 2.08
C20:5n3	13.52 ± 0.03^a^	11.94 ± 1.08^ab^	10.57 ± 0.84^b^	10.89 ± 0.50^b^	12.10 ± 0.45	10.99 ± 1.33	10.21 ± 1.02	11.78 ± 0.59
C22:5n3	0.18 ± 0.01	0.19 ± 0.01	0.18 ± 0.01	0.18 ± 0.01	0.99 ± 0.23	1.07 ± 0.12	1.03 ± 0.40	1.09 ± 0.14
C22:6n3	3.05 ± 0.06	2.72 ± 0.06	2.70 ± 0.29	2.78 ± 0.12	5.74 ± 0.03	5.20 ± 0.20	4.96 ± 0.57	4.85 ± 0.06
∑PUFA	38.26 ± 0.19	37.25 ± 1.49	35.38 ± 1.28	36.95 ± 1.71	45.04 ± 0.37	43.92 ± 2.26	42.87 ± 2.66	46.24 ± 2.60
∑n-3PUFA	17.76 ± 0.05^a^	16.00 ± 1.11^ab^	14.50 ± 1.10^b^	15.02 ± 0.59^ab^	20.07 ± 0.17	18.60 ± 1.56	17.23 ± 1.98	19.06 ± 0.41
∑n-6PUFA	20.51 ± 0.14	21.24 ± 0.38	20.88 ± 0.17	21.93 ± 1.12	24.97 ± 0.21	25.32 ± 0.70	25.64 ± 0.68	27.18 ± 2.19
n-3/n-6	0.87 ± 0.01^a^	0.76 ± 0.07^ab^	0.69 ± 0.05^b^	0.69 ± 0.02^b^	0.80 ± 0.01	0.74 ± 0.05	0.67 ± 0.06	0.70 ± 0.04
∑LC-PUFA	28.39 ± 0.14	27.54 ± 2.13	25.19 ± 1.60	25.64 ± 2.38	33.87 ± 1.08	32.71 ± 2.85	32.41 ± 2.97	35.32 ± 3.43
Unknown	6.53 ± 0.24	7.91 ± 1.63	6.64 ± 0.33	6.20 ± 0.24	9.54 ± 1.26	9.19 ± 0.54	9.03 ± 0.88	7.60 ± 1.15

Data are presented as mean ± SE (n = 2). Values within the same row with different letters mean significant difference. Fatty acids contents < 0.5% are not listed in this table. ∑SFA: total saturated fatty acids; ∑MUFA: total monounsaturated fatty acids; ∑PUFA: total polyunsaturated fatty acids; ∑LC-PUFA: total long chain polyunsaturated fatty acids.

**Fig 2:**
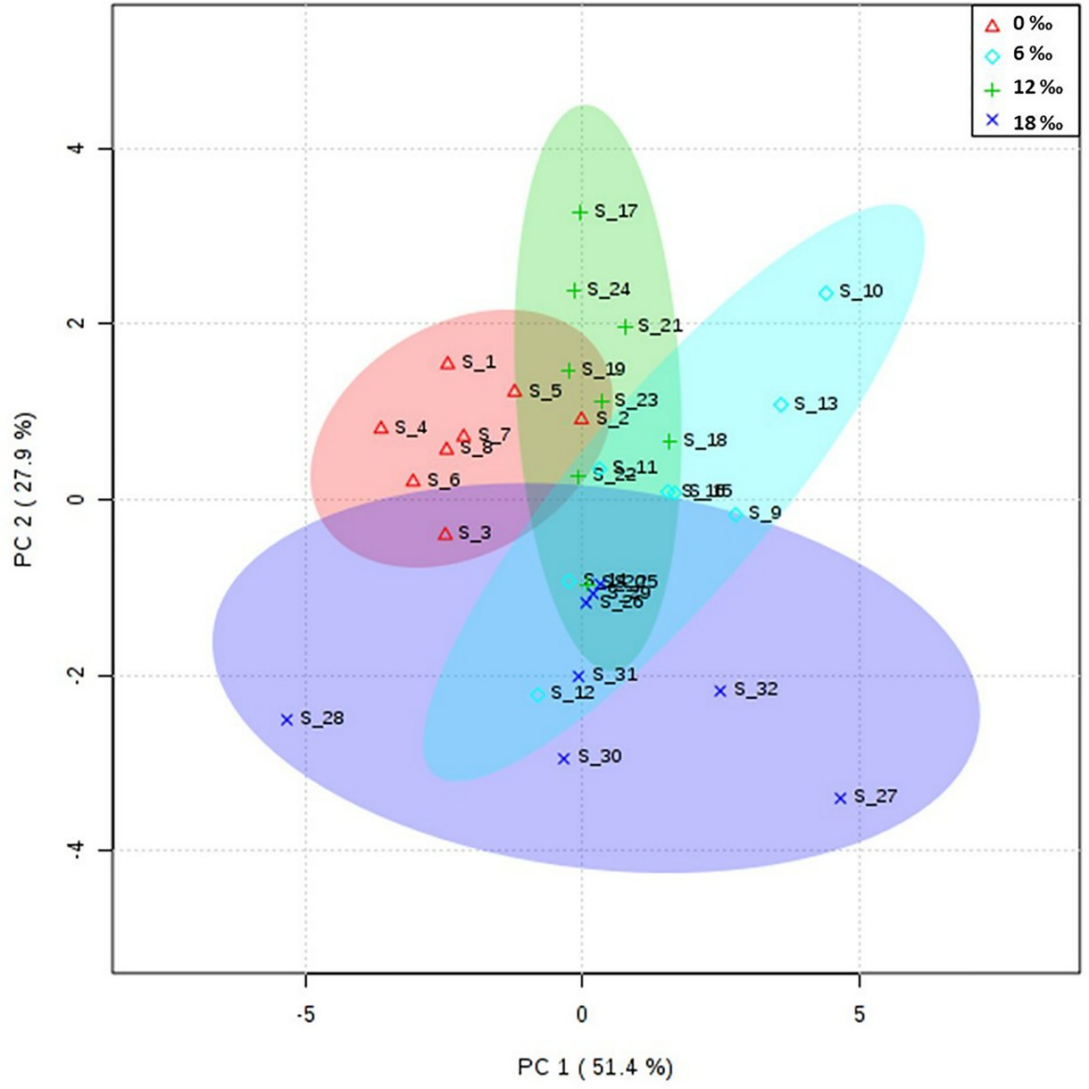
PCA scores plot of fatty acids profiles in the gonads of adult female *E*. *sinensis*.

The results of PCA showed that the fatty acid profile in the ovaries of female crabs in the 0‰ treatment group was similar to those of the 6‰ and 12‰ treatment groups; however, they were slightly different from that in the 18‰ treatment group (Fig 2). For the fatty acid composition of the neutral lipids, the female crabs in the 18‰ treatment group had the highest percentage of C18:1n9, while the highest percentage of C22:6n3 (DHA) was detected in the 0‰ treatment group (*P* < 0.05, Table 3). For the polar lipids, the percentage of C18:1n9 showed an increasing trend with increasing salinity (*P* < 0.05), while no significant differences were found for the other fatty acids among four salinity treatments (*P* > 0.05).

**Table 3:** The principal fatty acid profiles (% total fatty acids) of the neutral lipids and polar lipids in the ovaries of adult female *E*. *sinensis*.

Fatty acids	Neutral lipids				Polar lipids			
	0 ‰	6 ‰	12 ‰	18 ‰	0 ‰	6 ‰	12 ‰	18 ‰
C14:0	1.08 ± 0.02	1.05 ± 0.06	1.07 ± 0.07	0.94 ± 0.01	0.34 ± 0.01	0.33 ± 0.02	0.32 ± 0.02	0.29 ± 0.01
C16:0	14.90 ± 0.03	14.60 ± 0.14	14.41 ± 0.26	14.76 ± 0.21	8.83 ± 0.14	8.89 ± 0.01	8.44 ± 0.24	9.28 ± 0.27
C18:0	3.21 ± 0.01	3.29 ± 0.17	3.25 ± 0.05	3.28 ± 0.04	5.76 ± 0.16	6.03 ± 0.48	5.90 ± 0.10	5.72 ± 0.22
∑SFA	19.00 ± 0.01	18.75 ± 0.34	18.58 ± 0.28	18.91 ± 0.24	15.24 ± 0.31	15.57 ± 0.49	15.01 ± 0.30	15.64 ± 0.48
C16:1	11.72 ± 0.49	11.44 ± 0.85	10.61 ± 0.49	12.08 ± 0.07	7.69 ± 0.28	7.24 ± 0.69	6.59 ± 0.13	7.81 ± 0.28
C17:1n7	0.76 ± 0.01	0.71 ± 0.01	0.77 ± 0.01	0.75 ± 0.05	0.57 ± 0.03	0.57 ± 0.03	0.62 ± 0.03	0.62 ± 0.01
C18:1n9	22.82 ± 0.07^b^	23.32 ± 0.34^ab^	23.68 ± 0.11^ab^	24.27 ± 0.17^a^	20.55 ± 0.20^b^	20.99 ± 0.23^ab^	21.27 ± 0.32^ab^	22.40 ± 0.47^a^
C18:1n7	5.29 ± 0.15	5.20 ± 0.34	5.12 ± 0.27	5.32 ± 0.01	6.35 ± 0.39	6.33 ± 0.42	6.21 ± 0.45	6.59 ± 0.03
C20:1n9	0.56 ± 0.01	0.52 ± 0.02	0.59 ± 0.05	0.55 ± 0.01	0.86 ± 0.03	0.88 ± 0.06	1.02 ± 0.06	0.87 ± 0.04
∑MUFA	41.14 ± 0.43	41.18 ± 0.88	40.75 ± 0.71	42.97 ± 0.28	36.02 ± 0.14	35.99 ± 1.40	35.69 ± 0.99	38.28 ± 0.82
C18:2n6	9.74 ± 0.58	12.26 ± 1.40	11.20 ± 0.51	10.35 ± 0.50	9.21 ± 0.78	11.02 ± 1.05	10.17 ± 0.29	9.78 ± 0.31
C18:3n3	1.82 ± 0.09	2.15 ± 0.01	2.11 ± 0.21	2.03 ± 0.19	1.52 ± 0.02	1.90 ± 0.03	1.91 ± 0.20	1.86 ± 0.16
C20:2n6	1.06 ± 0.01	1.17 ± 0.06	1.19 ± 0.03	1.15 ± 0.01	1.22 ± 0.01	1.31 ± 0.03	1.36 ± 0.02	1.36 ± 0.03
C20:4n6	3.52 ± 0.09	3.49 ± 0.18	3.58 ± 0.40	3.46 ± 0.05	5.63 ± 0.13	5.48 ± 0.03	5.53 ± 0.65	5.16 ± 0.22
C20:5n3	6.82 ± 0.25	6.08 ± 0.16	6.63 ± 0.02	6.18 ± 0.07	10.78 ± 0.22	10.38 ± 0.16	10.72 ± 0.06	10.05 ± 0.22
C22:5n3	0.74 ± 0.03	0.67 ± 0.01	0.70 ± 0.03	0.69 ± 0.04	1.62 ± 0.12	1.62 ± 0.05	1.54 ± 0.09	1.57 ± 0.13
C22:6n3	7.55 ± 0.17^a^	5.88 ± 0.21^b^	6.78 ± 0.28^ab^	5.77 ± 0.12^b^	10.57 ± 0.05	9.52 ± 0.01	10.34 ± 0.09	9.21 ± 0.65
∑PUFA	31.55 ± 0.64	32.01 ± 0.89	32.53 ± 0.95	29.98 ± 0.57	40.88 ± 0.69	41.59 ± 1.20	41.90 ± 0.84	39.37 ± 1.37
∑n-3PUFA	17.23 ± 0.16^a^	15.10 ± 0.38^b^	16.56 ± 0.02^a^	15.01 ± 0.02^b^	24.82 ± 0.04	23.79 ± 0.15	24.85 ± 0.11	23.07 ± 0.82
∑n-6PUFA	14.32 ± 0.48	16.91 ± 1.27	15.97 ± 0.94	14.97 ± 0.56	16.06 ± 0.65	17.80 ± 1.04	17.06 ± 0.95	16.30 ± 0.56
n-3/n-6	1.21 ± 0.07	0.90 ± 0.05	1.04 ± 0.04	1.04 ± 0.13	1.55 ± 0.06	1.34 ± 0.05	1.46 ± 0.06	1.45 ± 0.16
∑LC-PUFA	18.93 ± 0.03	16.44 ± 0.55	18.02 ± 0.62	16.44 ± 0.25	28.93 ± 0.06	27.37 ± 0.12	28.47 ± 0.65	26.38 ± 1.06
Unknown	8.31 ± 0.19	8.05 ± 0.36	8.15 ± 0.03	8.16 ± 0.05	7.87 ± 0.24	6.85 ± 0.29	7.40 ± 0.46	6.71 ± 0.06

Data are presented as mean ± SE (n = 2). Values within the same row with different letters mean significant difference. Fatty acids contents < 0.5% are not listed in this table. ∑SFA: total saturated fatty acids; ∑MUFA: total monounsaturated fatty acids; ∑PUFA: total polyunsaturated fatty acids; ∑LC-PUFA: total long chain polyunsaturated fatty acids.

### Fatty acid profiles of the hepatopancreas

**Fig 3:**
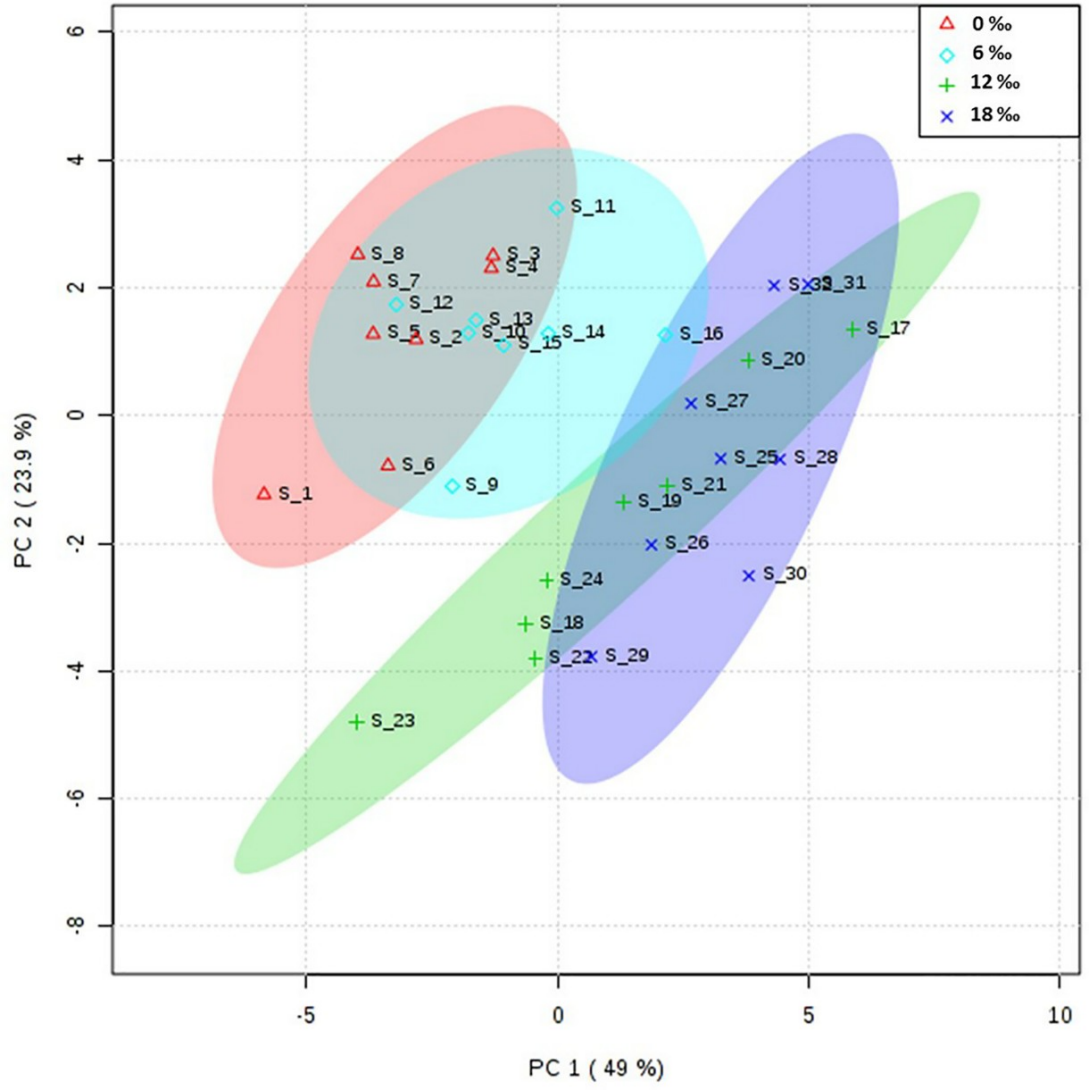
PCA scores plot of fatty acids profiles in the hepatopancreas of adult male *E*. *sinensis*.

The results of PCA showed that the fatty acid profile in the hepatopancreas of male crabs from the 0‰ treatment group was significantly different from that in the 12‰ and 18‰ treatment groups (Fig 3). For the fatty acid composition of the neutral lipids, there were no significant differences in the percentages of ∑SFA and most monounsaturated fatty acids among all treatments (*P* > 0.05, Table 4). The percentage of ∑MUFA in the neutral lipids increased significantly with increasing salinity (*P* < 0.05). The percentages of EPA, DHA, ∑n-3PUFA, and ∑LC-PUFA as well as n-3/n-6 PUFA ratio decreased significantly with increasing salinity (*P* < 0.05). For the polar lipids, the percentage of C20:2n6 increased significantly with increasing salinity, while the levels of DHA and ∑n-3PUFA showed a decreasing trend with increasing salinity (*P* < 0.05).

**Table 4:** The principal fatty acid profiles (% total fatty acids) of the neutral lipids and polar lipids in the hepatopancreas of adult male *E*. *sinensis*.

Fatty acids	Neutral lipids				Polar lipids			
	0 ‰	6 ‰	12 ‰	18 ‰	0 ‰	6 ‰	12 ‰	18 ‰
C14:0	2.14 ± 0.02	1.95 ± 0.04	2.00 ± 0.19	1.81 ± 0.06	2.25 ± 0.08	2.07 ± 0.13	1.89 ± 0.12	1.91 ± 0.18
C16:0	17.85 ± 0.81	17.90 ± 0.27	18.36 ± 0.05	18.02 ± 0.41	19.72 ± 0.50	21.88 ± 0.91	20.63 ± 0.56	21.77 ± 1.27
C18:0	3.87 ± 0.10	3.60 ± 0.18	3.62 ± 0.09	3.64 ± 0.08	4.50 ± 0.47	4.86 ± 0.55	5.09 ± 0.32	4.57 ± 0.29
∑SFA	26.47 ± 0.95	25.99 ± 0.57	26.86 ± 0.20	26.08 ± 0.38	28.52 ± 1.51	30.89 ± 0.30	29.79 ± 0.04	30.47 ± 1.14
C16:1	8.88 ± 0.08	9.56 ± 0.07	9.21 ± 0.06	9.17 ± 0.07	9.72 ± 0.45	10.42 ± 0.25	9.69 ± 0.07	9.87 ± 0.10
C17:1n7	0.73 ± 0.02	0.70 ± 0.02	0.77 ± 0.01	0.73 ± 0.03	0.68 ± 0.02^a^	0.52 ± 0.01^b^	0.63 ± 0.05^b^	0.59 ± 0.03^b^
C18:1n9	25.20 ± 0.19	25.70 ± 0.38	25.71 ± 0.04	26.56 ± 0.08	17.12 ± 0.75	15.61 ± 0.42	17.26 ± 0.85	16.96 ± 0.69
C18:1n7	3.70 ± 0.09	3.67 ± 0.08	3.85 ± 0.06	3.90 ± 0.02	3.82 ± 0.02	3.92 ± 0.04	4.31 ± 0.04	3.89 ± 0.17
C20:1n9	1.51 ± 0.01^a^	1.23 ± 0.01^b^	1.48 ± 0.01^a^	1.49 ± 0.05^a^	0.89 ± 0.02^a^	0.65 ± 0.03^b^	0.81 ± 0.01^a^	0.80 ± 0.01^a^
∑MUFA	40.81 ± 0.33^b^	41.49 ± 0.38^ab^	41.73 ± 0.10^ab^	42.57 ± 0.07^a^	32.81 ± 1.26	31.57 ± 0.26	33.18 ± 0.81	32.62 ± 0.79
C18:2n6	11.06 ± 0.17	10.99 ± 0.25	9.83 ± 0.73	12.11 ± 0.01	8.43 ± 0.34	8.49 ± 0.61	8.05 ± 1.78	9.91 ± 0.23
C18:3n3	1.42 ± 0.01	1.53 ± 0.03	1.21 ± 0.15	1.53 ± 0.19	1.13 ± 0.02	1.22 ± 0.06	1.00 ± 0.16	1.27 ± 0.21
C20:2n6	1.27 ± 0.03^c^	1.44 ± 0.01^b^	1.56 ± 0.03^a^	1.63 ± 0.01^a^	0.89 ± 0.04^b^	0.97 ± 0.04^ab^	1.08 ± 0.02^ab^	1.12 ± 0.02^a^
C20:4n6	1.46 ± 0.05	1.70 ± 0.03	1.73 ± 0.18	1.46 ± 0.10	3.50 ± 0.03	4.24 ± 0.56	4.58 ± 0.15	4.07 ± 0.17
C20:5n3	2.33 ± 0.11^a^	2.16 ± 0.14^ab^	1.92 ± 0.22^ab^	1.65 ± 0.12^b^	5.87 ± 0.34	5.83 ± 0.34	5.46 ± 0.09	4.65 ± 0.39
C22:5n3	0.70 ± 0.02	0.66 ± 0.02	0.71 ± 0.09	0.57 ± 0.01	0.67 ± 0.03	0.70 ± 0.01	0.75 ± 0.05	0.65 ± 0.06
C22:6n3	4.87 ± 0.16^a^	4.19 ± 0.36^ab^	4.24 ± 0.58^ab^	3.36 ± 0.09^b^	6.90 ± 0.05^a^	6.44 ± 0.26^ab^	6.35 ± 0.26^ab^	5.73 ± 0.29^b^
∑PUFA	23.52 ± 0.23	23.12 ± 0.67	21.70 ± 0.76	22.77 ± 0.07	27.69 ± 0.18	28.20 ± 0.06	27.58 ± 1.43	27.72 ± 0.48
∑n-3PUFA	9.57 ± 0.30^a^	8.81 ± 0.48^ab^	8.39 ± 0.74^ab^	7.44 ± 0.01^b^	14.77 ± 0.23^a^	14.37 ± 0.02^ab^	13.75 ± 0.51^ab^	12.49 ± 0.54^b^
∑n-6PUFA	13.95 ± 0.07	14.31 ± 0.19	13.31 ± 0.50	15.33 ± 0.08	12.92 ± 0.41	13.82 ± 0.07	13.83 ± 1.67	15.23 ± 0.04
n-3/n-6	0.70 ± 0.03^a^	0.62 ± 0.03^ab^	0.65 ± 0.02^ab^	0.49 ± 0.01^b^	1.17 ± 0.06^a^	1.05 ± 0.01^ab^	1.02 ± 0.15^ab^	0.82 ± 0.03^b^
∑LC-PUFA	9.77 ± 0.37^a^	9.16 ± 0.46^ab^	9.09 ± 0.55^ab^	7.50 ± 0.28^b^	17.25 ± 0.22	17.51 ± 0.65	17.46 ± 0.54	15.42 ± 0.91
Unknown	9.20 ± 0.58	9.40 ± 0.35	9.72 ± 0.29	8.58 ± 0.46	10.71 ± 0.50	9.35 ± 0.48	9.45 ± 0.32	9.19 ± 0.44

Data are presented as mean ± SE (n = 2). Values within the same row with different letters mean significant difference. Fatty acids contents < 0.5% are not listed in this table. ∑SFA: total saturated fatty acids; ∑MUFA: total monounsaturated fatty acids; ∑PUFA: total polyunsaturated fatty acids; ∑LC-PUFA: total long chain polyunsaturated fatty acids.

**Fig 4:**
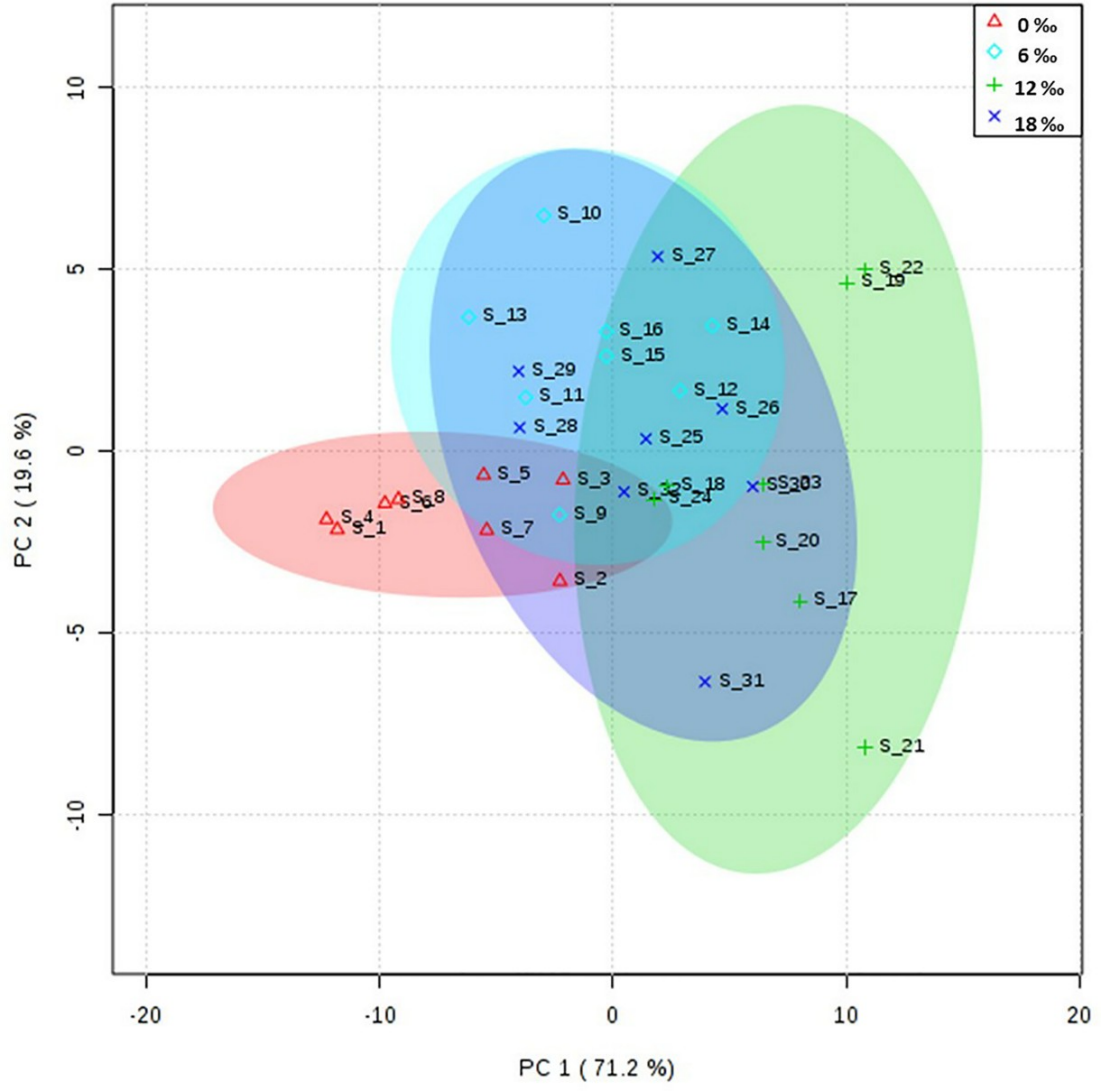
PCA scores plot of fatty acids profiles in the hepatopancreas of adult female *E*. *sinensis*.

The results of PCA showed that the fatty acid profile in the hepatopancreas of female crabs from the 0‰ treatment group was similar to those of the 6‰ and 18‰ treatment groups, but different from that of the 12‰ treatment group (Fig 4). The fatty acid composition in the neutral lipids and polar lipids of the hepatopancreas are shown in Table 5. For the neutral lipids, the highest percentages of C18:0 and ∑SFA were detected in the 0‰ treatment group, while the highest percentages of EPA, ∑n-3PUFA, ∑n-6PUFA, ∑PUFA, and ∑LC-PUFA were detected in the 12‰ treatment group (*P* < 0.05). For the polar lipids, the 0‰ salinity group had the highest percentages of most saturated fatty acids and monounsaturated fatty acids, while the highest levels of C18:2n6, C18:3n3, C20:4n6, EPA, DHA, ∑PUFA, ∑n-3PUFA, ∑n-6PUFA and ∑LC-PUFA were detected in the 12‰ treatment group (*P* < 0.05).

**Table 5:** The principal fatty acid profiles (% total fatty acids) of the neutral lipids and polar lipids in the hepatopancreas of adult female *E*. *sinensis*.

Fatty acids	Neutral lipids				Polar lipids			
	0 ‰	6 ‰	12 ‰	18 ‰	0 ‰	6 ‰	12 ‰	18 ‰
C14:0	1.91 ± 0.17	1.75 ± 0.04	1.71 ± 0.04	1.80 ± 0.21	1.86 ± 0.04	1.68 ± 0.01	1.58 ± 0.08	1.69 ± 0.03
C16:0	19.7 ± 0.84	18.55 ± 0.09	17.72 ± 0.22	18.08 ± 0.69	22.85 ± 0.25^a^	21.48 ± 0.15^ab^	19.85 ± 0.04^b^	20.33 ± 0.02^ab^
C18:0	3.82 ± 0.17^a^	3.44 ± 0.17^ab^	3.10 ± 0.06^b^	3.62 ± 0.07^ab^	3.73 ± 0.04	3.68 ± 0.05	3.87 ± 0.05	3.79 ± 0.03
∑SFA	28.32 ± 1.21^a^	26.40 ± 0.18^ab^	24.92 ± 0.12^b^	26.29 ± 0.72^ab^	30.76 ± 0.81^a^	28.86 ± 0.35^ab^	26.95 ± 0.09^b^	27.75 ± 0.22^ab^
C16:1n7	9.98 ± 0.57	9.85 ± 0.47	9.83 ± 0.28	9.87 ± 0.08	11.77 ± 0.03	11.37 ± 0.54	10.79 ± 0.02	11.32 ± 0.59
C17:1n7	0.71 ± 0.02	0.73 ± 0.01	0.71 ± 0.01	0.77 ± 0.05	0.64 ± 0.02^a^	0.57 ± 0.01^ab^	0.51 ± 0.02^b^	0.55 ± 0.03^b^
C18:1n9	26.67 ± 0.22	26.91 ± 0.43	25.81 ± 0.61	26.16 ± 1.07	24.42 ± 0.72	24.46 ± 0.50	22.43 ± 0.28	23.34 ± 0.57
C18:1n7	4.73 ± 0.04	4.51 ± 0.22	4.35 ± 0.17	4.57 ± 0.11	4.60 ± 0.04^a^	4.28 ± 0.02^ab^	4.07 ± 0.11^b^	4.38 ± 0.07^ab^
C20:1n9	1.48 ± 0.13	1.36 ± 0.03	1.19 ± 0.08	1.34 ± 0.14	0.79 ± 0.12^a^	0.72 ± 0.02^a^	0.56 ± 0.01^b^	0.68 ± 0.04^ab^
∑MUFA	44.23 ± 0.79	43.95 ± 0.19	42.44 ± 1.00	43.34 ± 2.20	42.70 ± 0.54^a^	41.93 ± 0.10^ab^	38.83 ± 0.30^b^	40.83 ± 0.05^ab^
C18:2n6	8.08 ± 1.31	10.67 ± 0.31	10.56 ± 0.18	9.36 ± 0.30	5.95 ± 0.64^b^	8.58 ± 0.10^a^	8.80 ± 0.10^a^	8.11 ± 0.16^a^
C18:3n3	0.88 ± 0.08	1.17 ± 0.12	1.31 ± 0.13	1.26 ± 0.09	0.54 ± 0.06^b^	0.79 ± 0.09^a^	0.90 ± 0.11^a^	0.76 ± 0.03^a^
C20:2n6	1.70 ± 0.06	1.75 ± 0.03	1.76 ± 0.02	1.66 ± 0.29	1.00 ± 0.03	0.99 ± 0.05	1.06 ± 0.01	0.96 ± 0.06
C20:4n6	1.21 ± 0.07	1.20 ± 0.07	1.60 ± 0.16	1.27 ± 0.13	1.53 ± 0.11^b^	1.83 ± 0.11^b^	2.66 ± 0.12^a^	1.88 ± 0.07^b^
C20:5n3	1.37 ± 0.17^ab^	1.15 ± 0.08^b^	1.86 ± 0.12^a^	1.35 ± 0.26^ab^	2.27 ± 0.01^b^	2.09 ± 0.19^b^	3.46 ± 0.19^a^	2.59 ± 0.27^b^
C22:6n3	2.71 ± 0.38	2.09 ± 0.13	3.86 ± 0.09	2.98 ± 0.46	2.70 ± 0.19^b^	2.52 ± 0.01^b^	4.29 ± 0.13^a^	3.17 ± 0.57^b^
∑PUFA	16.43 ± 1.07^b^	18.54 ± 0.56^ab^	21.48 ± 0.21^a^	18.37 ± 1.63^ab^	14.21 ± 0.98^c^	17.06 ± 0.44^b^	21.48 ± 0.19^a^	17.73 ± 0.74^b^
∑n-3PUFA	5.27 ± 0.63^b^	4.75 ± 0.37^b^	7.37 ± 0.19^a^	5.91 ± 0.49^ab^	5.67 ± 0.25^b^	5.57 ± 0.28^b^	8.87 ± 0.18^a^	6.69 ± 0.88^b^
∑n-6PUFA	11.16 ± 0.43^b^	13.79 ± 0.19^ab^	14.11 ± 0.02^a^	12.46 ± 0.14^ab^	8.54 ± 0.73^b^	11.50 ± 0.16^a^	12.62 ± 0.02^a^	11.04 ± 0.14^a^
n-3/n-6	0.48 ± 0.02^ab^	0.35 ± 0.03^b^	0.54 ± 0.06^a^	0.49 ± 0.07^ab^	0.67 ± 0.02^ab^	0.49 ± 0.01^b^	0.72 ± 0.01^a^	0.61 ± 0.08^ab^
∑LC-PUFA	5.77 ± 0.61^ab^	4.95 ± 0.17^b^	7.85 ± 0.24^a^	6.09 ± 0.52^ab^	6.73 ± 0.29^b^	6.71 ± 0.29^b^	10.72 ± 0.18^a^	7.90 ± 0.93^b^
Unknown	11.02 ± 0.36	11.12 ± 0.45	11.16 ± 0.28	12.00 ± 0.55	12.32 ± 0.43	12.15 ± 0.60	12.74 ± 0.52	12.22 ± 0.47

Data are presented as mean ± SE (n = 2). Values within the same row with different letters mean significant difference. Fatty acids contents < 0.5% are not listed in this table. ∑SFA: total saturated fatty acids; ∑MUFA: total monounsaturated fatty acids; ∑PUFA: total polyunsaturated fatty acids; ∑LC-PUFA: total long chain polyunsaturated fatty acids.

### Fatty acid profiles of muscles

**Fig 5:**
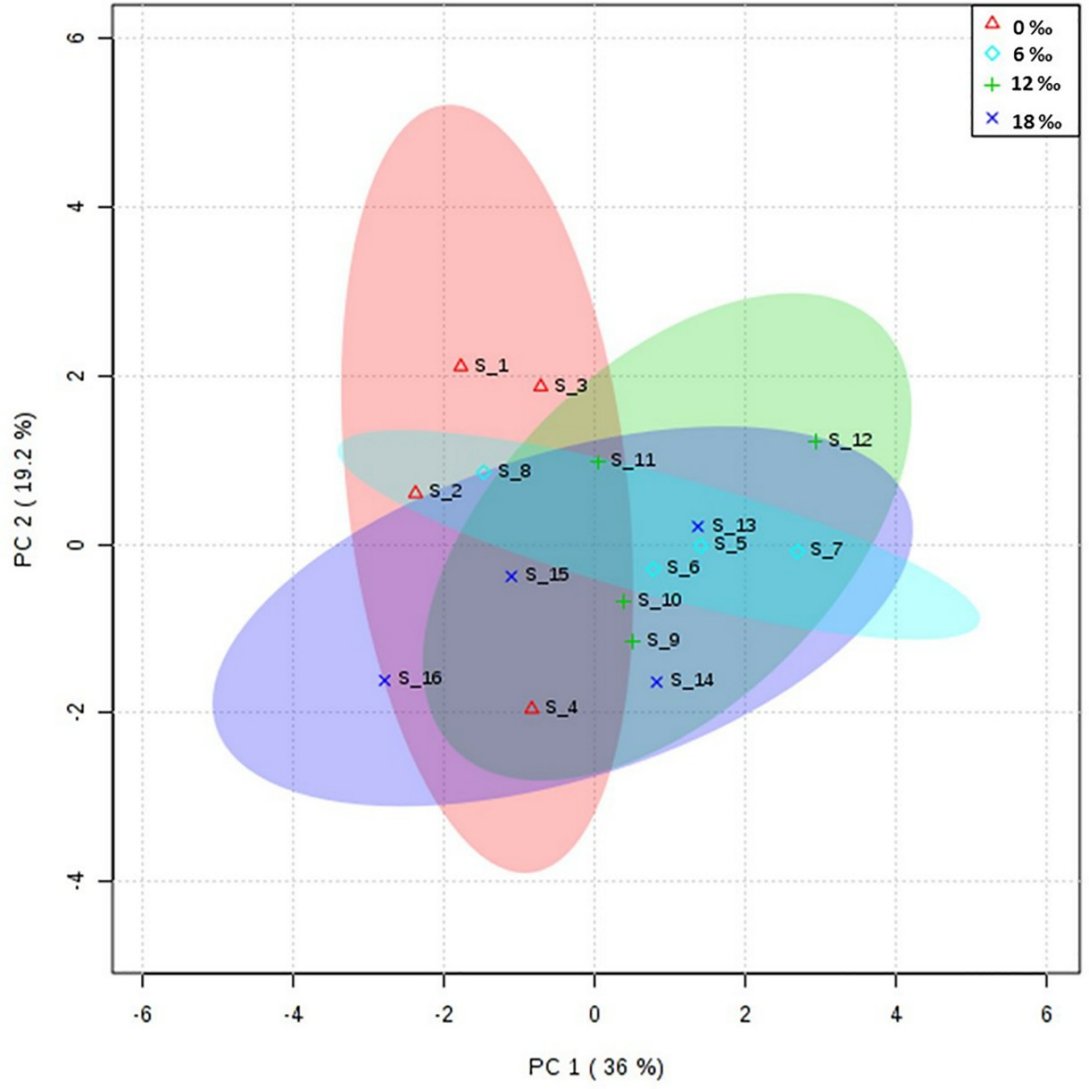
PCA scores plot of fatty acids profiles in the muscles of adult male *E*. *sinensis*.

The Fig 5 shows that the fatty acids profiles in the muscles of male crabs were similar among all treatments. The fatty acid composition of male muscles is shown in Table 6. For the neutral lipids, the percentages of C16:0 and ∑SFA showed an overall decreasing trend with increasing salinity (*P* < 0.05), while no significant differences were found for the percentages of most monounsaturated fatty acids and polyunsaturated fatty acids. For the polar lipids, the highest C18:0 was detected in the 18‰ treatment group (*P* < 0.05). The males from the 12‰ treatment group had the highest percentage of C18:1n7 in the polar lipids (*P* < 0.05), while no significant differences were found for the other monounsaturated fatty acids among all treatments. The highest percentage of C22:6n3 showed an overall decreasing trend with increasing salinity (*P* < 0.05), while no significant differences were found for the other polyunsaturated fatty acids among four salinity treatments.

**Table 6:** The principal fatty acid profiles (% total fatty acids) of the neutral lipids and polar lipids in the muscles of adult male *E*. *sinensis*.

Fatty acids	Neutral lipids				Polar lipids			
	0 ‰	6 ‰	12 ‰	18 ‰	0 ‰	6 ‰	12 ‰	18 ‰
C16:0	9.38 ± 0.28^a^	9.02 ± 0.09^ab^	8.82 ± 0.03^ab^	8.77 ± 0.06^b^	11.20 ± 0.10	10.82 ± 0.08	10.53 ± 0.53	11.05 ± 0.68
C18:0	11.11 ± 0.09	10.96 ± 0.08	10.72 ± 0.21	11.01 ± 0.05	6.03 ± 0.06^b^	6.50 ± 0.13^ab^	6.13 ± 0.05^ab^	6.76 ± 0.30^a^
∑SFA	21.42 ± 0.37^a^	20.80 ± 0.01^ab^	20.40 ± 0.21^b^	20.59 ± 0.03^ab^	18.31 ± 0.10	18.32 ± 0.23	17.71 ± 0.55	18.83 ± 1.02
C16:1	1.85 ± 0.13	2.21 ± 0.05	2.11 ± 0.14	1.91 ± 0.05	2.70 ± 0.02	2.68 ± 0.10	2.77 ± 0.21	2.62 ± 0.14
C18:1n9	15.69 ± 0.33	15.90 ± 0.44	16.03 ± 0.43	16.53 ± 0.23	20.37 ± 0.03	19.85 ± 0.11	20.19 ± 0.70	20.64 ± 0.52
C18:1n7	3.35 ± 0.02	3.65 ± 0.01	3.77 ± 0.21	3.68 ± 0.28	3.55 ± 0.11^b^	3.93 ± 0.09^ab^	4.27 ± 0.17^a^	4.09 ± 0.27^ab^
C20:1n9	0.89 ± 0.13	0.75 ± 0.03	0.85 ± 0.05	0.91 ± 0.11	0.62 ± 0.04	0.61 ± 0.03	0.72 ± 0.03	0.62 ± 0.06
∑MUFA	22.13 ± 0.37	22.85 ± 0.46	23.13 ± 0.52	23.37 ± 0.24	27.75 ± 0.25	27.54 ± 0.34	28.48 ± 0.64	28.48 ± 0.89
C18:2n6	11.95 ± 0.81	11.56 ± 0.70	10.72 ± 0.49	12.03 ± 0.38	9.77 ± 0.85	8.59 ± 0.21	8.65 ± 0.48	9.18 ± 0.02
C18:3n3	1.28 ± 0.05	1.47 ± 0.11	1.18 ± 0.04	1.47 ± 0.11	0.89 ± 0.05	0.94 ± 0.05	0.81 ± 0.02	0.99 ± 0.10
C20:2n6	2.99 ± 0.22	2.96 ± 0.21	3.25 ± 0.18	3.11 ± 0.06	1.39 ± 0.07	1.46 ± 0.03	1.48 ± 0.04	1.50 ± 0.17
C20:4n6	6.88 ± 0.12	8.01 ± 0.08	7.97 ± 0.16	7.28 ± 0.92	5.22 ± 0.07	6.60 ± 0.02	6.42 ± 0.38	5.93 ± 0.81
C20:5n3	16.84 ± 0.97	16.88 ± 0.24	16.61 ± 0.15	16.87 ± 0.02	17.78 ± 0.57	18.14 ± 0.26	17.36 ± 0.41	17.80 ± 0.02
C22:5n3	0.47 ± 0.01^b^	0.52 ± 0.02^ab^	0.58 ± 0.01^a^	0.54 ± 0.04^ab^	0.71 ± 0.04	0.80 ± 0.02	0.84 ± 0.04	0.75 ± 0.09
C22:6n3	12.74 ± 0.10	11.96 ± 0.53	12.63 ± 0.06	11.75 ± 0.62	13.66 ± 0.07^a^	13.33 ± 0.19^ab^	13.48 ± 0.28^ab^	12.65 ± 0.34^b^
∑PUFA	53.66 ± 0.14	53.92 ± 0.09	53.54 ± 0.42	53.67 ± 0.16	49.59 ± 0.11	50.06 ± 0.25	49.25 ± 0.68	49.01 ± 1.54
∑n-3PUFA	31.84 ± 0.77	31.39 ± 0.91	31.61 ± 0.10	31.26 ± 0.44	33.21 ± 0.61	33.42 ± 0.02	32.70 ± 0.74	32.40 ± 0.56
∑n-6PUFA	21.82 ± 0.90	22.53 ± 0.82	21.93 ± 0.51	22.41 ± 0.60	16.38 ± 0.71	16.65 ± 0.26	16.55 ± 0.06	16.61 ± 0.98
n-3/n-6	1.46 ± 0.05	1.40 ± 0.04	1.45 ± 0.03	1.40 ± 0.04	2.03 ± 0.05	2.01 ± 0.06	1.98 ± 0.04	1.96 ± 0.05
∑LC-PUFA	37.44 ± 0.93	37.93 ± 0.89	38.39 ± 0.29	37.07 ± 0.37	37.55 ± 0.63	39.07 ± 0.06	38.31 ± 1.10	37.35 ± 1.27
Unknown	2.80 ± 0.13	2.67 ± 0.33	2.94 ± 0.31	2.38 ± 0.11	4.36 ± 0.24	4.08 ± 0.13	4.57 ± 0.49	3.88 ± 0.36

Data are presented as mean ± SE (n = 2). Values within the same row with different letters mean significant difference. Fatty acids contents < 0.5% are not listed in this table. ∑SFA: total saturated fatty acids; ∑MUFA: total monounsaturated fatty acids; ∑PUFA: total polyunsaturated fatty acids; ∑LC-PUFA: total long chain polyunsaturated fatty acids.

**Fig 6:**
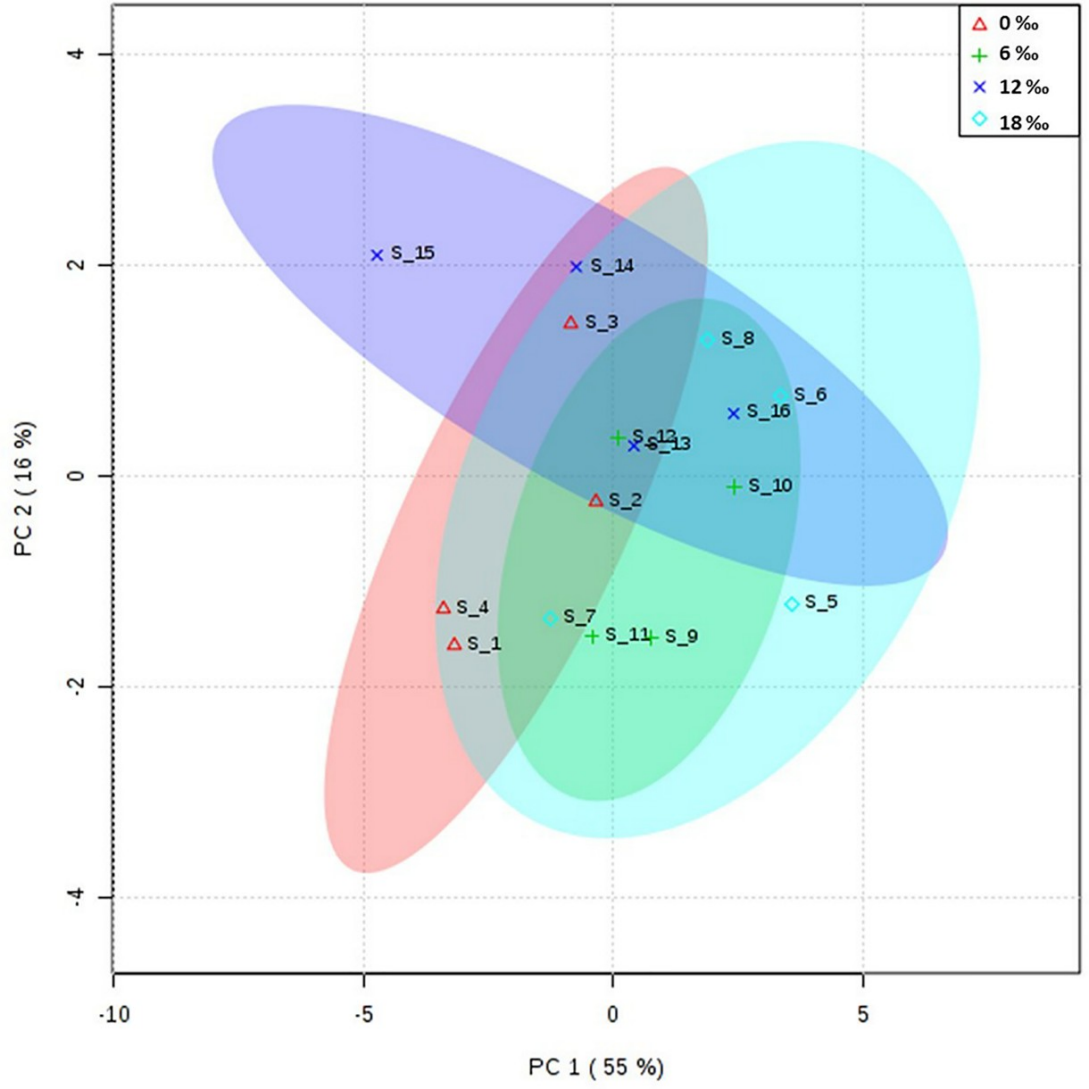
PCA scores plot of fatty acids profiles in the muscles of adult female *E. sinensis*.

The fatty acids profiles in the muscles of female crabs from the four treatment groups were similar (Fig 6). For the fatty acid composition in neutral lipids, there were no significant differences in the percentages of most saturated fatty acids, monounsaturated fatty acids, or polyunsaturated fatty acids among all treatments (*P* > 0.05, Table 7). For the polar lipids, the percentage of C18:1n9 increased with increasing salinity, while no significant differences were found for the percentages of most other monounsaturated fatty acids and ∑MUFA (*P* > 0.05). The 6‰ treatment group had the highest percentages of C18:3n3, and ∑n-6PUFA, while the highest levels of DHA, ∑n-3PUFA and ∑LC-PUFA were detected in the 0‰ treatment group (*P* < 0.05).

**Table 7:** The principal fatty acid profiles (% total fatty acids) of the neutral lipids and polar lipids in the muscles of adult female *E*. *sinensis*.

Fatty acids	Neutral lipids				Polar lipids			
	0 ‰	6 ‰	12 ‰	18 ‰	0 ‰	6 ‰	12 ‰	18 ‰
C16:0	9.51 ± 0.34	9.65 ± 0.08	9.51 ± 0.44	9.06 ± 0.02	10.96 ± 0.04	11.10 ± 0.03	11.09 ± 0.14	10.80 ± 0.02
C18:0	10.04 ± 0.27	10.04 ± 0.09	9.67 ± 0.17	10.12 ± 0.13	5.65 ± 0.11	5.38 ± 0.28	5.65 ± 0.08	5.48 ± 0.05
∑SFA	20.51 ± 0.65	20.56 ± 0.20	20.05 ± 0.26	19.99 ± 0.17	17.71 ± 0.10	17.53 ± 0.27	17.89 ± 0.30	17.30 ± 0.08
C16:1	2.70 ± 0.02	2.69 ± 0.13	2.48 ± 0.32	2.83 ± 0.06	3.46 ± 0.17	3.47 ± 0.34	3.22 ± 0.02	3.43 ± 0.07
C18:1n9	16.99 ± 0.27	17.28 ± 0.04	17.18 ± 0.89	18.04 ± 0.22	19.93 ± 0.23	20.25 ± 0.02	20.62 ± 0.06	20.91 ± 0.37
C18:1n7	3.61 ± 0.16	3.81 ± 0.29	3.60 ± 0.29	3.84 ± 0.06	4.08 ± 0.09	4.19 ± 0.20	4.15 ± 0.05	4.16 ± 0.10
C20:1n9	0.84 ± 0.02	0.86 ± 0.01	0.79 ± 0.05	0.94 ± 0.05	0.75 ± 0.01	0.74 ± 0.01	0.76 ± 0.05	0.79 ± 0.07
∑MUFA	24.49 ± 0.45	24.95 ± 0.43	24.40 ± 1.60	26.02 ± 0.39	28.75 ± 0.32	29.16 ± 0.57	29.32 ± 0.13	29.82 ± 0.60
C18:2n6	9.49 ± 0.33	10.78 ± 0.81	10.44 ± 0.34	9.69 ± 0.71	7.74 ± 0.53	10.29 ± 1.00	9.48 ± 0.67	8.10 ± 0.26
C18:3n3	0.95 ± 0.03	1.09 ± 0.03	1.09 ± 0.12	1.06 ± 0.01	0.83 ± 0.03^b^	1.13 ± 0.02^a^	1.06 ± 0.04^a^	0.94 ± 0.04^ab^
C20:2n6	2.52 ± 0.07	2.52 ± 0.12	2.56 ± 0.13	2.69 ± 0.08	1.64 ± 0.01	1.69 ± 0.02	1.73 ± 0.07	1.75 ± 0.03
C20:4n6	7.36 ± 0.16	7.64 ± 0.18	7.20 ± 0.61	8.09 ± 0.17	5.75 ± 0.36	5.76 ± 0.02	5.40 ± 0.25	6.09 ± 0.20
C20:5n3	15.82 ± 0.64	14.72 ± 0.27	14.70 ± 0.22	15.39 ± 0.03	16.82 ± 0.21^a^	15.43 ± 0.02^b^	15.52 ± 0.53^b^	16.10 ± 0.20^ab^
C22:5n3	0.66 ± 0.03^b^	0.83 ± 0.05^ab^	1.02 ± 0.13^a^	0.89 ± 0.10^ab^	1.07 ± 0.07	1.14 ± 0.03	1.12 ± 0.02	1.21 ± 0.05
C22:6n3	12.12 ± 0.37	10.92 ± 0.75	11.76 ± 0.13	11.39 ± 0.17	14.25 ± 0.03^a^	12.58 ± 0.56^b^	13.21 ± 0.33^ab^	13.07 ± 0.10^ab^
∑PUFA	49.42 ± 1.31	48.99 ± 0.56	49.27 ± 0.91	49.73 ± 0.69	48.37 ± 0.39	48.29 ± 0.42	47.80 ± 0.04	47.53 ± 0.70
∑n-3PUFA	30.06 ± 1.08	28.05 ± 1.07	29.07 ± 0.17	29.28 ± 0.27	33.23 ± 0.22^a^	30.55 ± 0.58^b^	31.19 ± 0.95^ab^	31.60 ± 0.27^ab^
∑n-6PUFA	19.37 ± 0.23	20.93 ± 0.52	20.20 ± 1.07	20.45 ± 0.95	15.14 ± 0.17	17.74 ± 1.00	16.61 ± 0.99	15.93 ± 0.43
n-3/n-6	1.56 ± 0.08	1.35 ± 0.10	1.45 ± 0.06	1.45 ± 0.13	2.21 ± 0.12^a^	1.74 ± 0.10^b^	1.89 ± 0.09^ab^	2.00 ± 0.12^ab^
∑LC-PUFA	36.47 ± 0.89	34.59 ± 1.21	35.18 ± 0.55	36.30 ± 0.11	38.15 ± 0.17^a^	35.18 ± 0.59^b^	35.53 ± 0.67^b^	36.75 ± 0.52^ab^
Unknown	4.50 ± 1.32	5.51 ± 0.78	5.31 ± 0.03	4.27 ± 0.13	5.17 ± 0.17	5.03 ± 0.12	5.00 ± 0.22	5.36 ± 0.03

Data are presented as mean ± SE (n = 2). Values within the same row with different letters mean significant difference. Fatty acids contents < 0.5% are not listed in this table. ∑SFA: total saturated fatty acids; ∑MUFA: total monounsaturated fatty acids; ∑PUFA: total polyunsaturated fatty acids; ∑LC-PUFA: total long chain polyunsaturated fatty acids.

## Discussion

### Effects of long-term salinity adaptation on lipid contents

Neutral lipids (mainly triglycerides) are important energy sources in crustaceans [13, 29], while polar lipids (mainly phospholipids) are the main structural components of membranes, which play important roles in maintaining membrane fluidity and permeability [15]. In this study, there was no significant difference in the contents of total lipids, neutral lipids, and polar lipids in the gonads of male crabs among the four salinity treatments after 40 days of salinity adaptation. Such results could be explained by the fact that the gonad is an important reproductive organ for male crabs [28], and therefore, its lipid content is maintained at a relatively stable level [37] to ensure normal reproductive and is thus not susceptible to fluctuating salinity. For female crabs, significantly higher polar lipids contents in the ovaries were measured at 0‰ treatment compared to that in the 18‰ group. Possible explanations for such results are: 1) The osmolality difference between the body of the female crabs and water in the 0‰ treatment was significantly greater than that of the other treatments [9, 10]; therefore, increasing polar lipids may improve the fluidity and permeability of membranes, which will promote ion transport, and maintain the osmotic and ionic balance [13, 38]. 2) The decrease of the polar lipids contents in the ovaries of females from high salinity treatment may be due to a direct effect of the increasing salinity, which is known to accelerate ovarian development and maturation [10], resulted in the lower contents of polar lipids in this treatment. Indeed, our recent result showed that female *E*. *sinensis* generally decreased the ovarian polar lipids contents and increased the contents of neutral lipids during ovarian maturation (our unpublished data).

The hepatopancreas is an important organ for lipid storage in *E*. *sinensis* [39]. Our results showed that the total lipids and neutral lipids contents in the hepatopancreas of males and females from the 6‰, 12‰, and 18‰ treatment groups were significantly higher than those in the 0‰ treatment group after 40 days of salinity adaptation. This could be because the osmolality of brackish water is closer to that in the body of the *E*. *sinensis*, hence reduced energy consumption is required for osmoregulation [9, 10], which is beneficial to the accumulation of energetic lipids (mainly neutral lipids) in the hepatopancreas. This is not consistent with a previous report [24], in which short-term salinity adaptation had no significant effects on the contents of total lipids and neutral lipids (mainly triglycerides) in the hepatopancreas of adult *E*. *sinensis*. The difference might be because the study of Chapelle was conducted for a short-term (3 or 14 days), and the energy consumption for osmoregulation was somewhat lower; therefore, there were no significant changes in the total lipid and neutral lipid contents in the hepatopancreas. In the present study, the polar lipids contents in the hepatopancreas of female crabs decreased with increasing salinity. This might be because polar lipids are important structural components of membranes, and increased phospholipid contents can increase the fluidity of membranes and maintain their integrity, which would facilitate ionic transport under low salinity conditions [38, 40]. However, previous experiments indicated that short-term salinity adaptation had no significant effects on the phospholipid content in the hepatopancreas of *E*. *sinensis* [24, 41] and the white Pacific shrimp *Litopenaeus vannamei* [42]. These discrepancies might reflect the fact that in these previous studies, the salinity adaptation time was too short to significantly change the phospholipid metabolism in the hepatopancreas.

The present study showed that the total lipids and polar lipids contents in the muscles of male and female crabs increased with increasing salinity. A possible explanation for this result was that increasing salinity promoted the gonadal development and maturation of *E*. *sinensis* [9, 10], which will induce estrus and higher level of activity [32]. Indeed, the activity of adult *E*. *sinensis* in the high-salinity treatment was greater compared to that in low salinity in this study. Zhuang et al. (2012) [43] also found that the activity frequency of adult *E*. *sinensis* was higher under high salinity conditions. Muscle is an important motor organ of *E*. *sinensis*, and the increasing polar lipids contents in the muscles might serve to maintain the normal physiological function of the membrane to ensure the normal movement for estrus chasing and mating during reproduction. Moreover, the content of neutral lipids in the muscles of female crabs decreased with increasing salinity, which might be because of the increasing salinity enhanced the movement frequency and increased energy consumption [10, 43]; therefore, the content of energy lipids (neutral lipids) in the muscles decreased with increasing salinity.

### Effects of long-term salinity adaptation on the fatty acid profiles

In this study, the fatty acid composition of neutral lipids and polar lipids in the gonads of male crabs were only limitedly affected by salinity, which might be because the gonad is an important reproductive organ for male crabs [28, 44]. The relatively stable fatty acid composition in the gonad of males is likely to ensure normal reproduction, hence the fatty acid compositions was not significantly affected by salinity. For the female crabs, the results of principal component analysis showed that the fatty acid composition in the ovaries from the 0‰ treatment group was similar to that of the 6‰ and 12‰ treatment groups, while a greater difference was found between the 0‰ and 18‰ treatment groups. The highest percentages of ∑MUFA in the neutral lipids and polar lipids in ovaries, as well as the C16:0 in polar lipids, were detected in the 18‰ treatment group, which indicated that saturated fatty acids and monounsaturated fatty acids were preferentially utilized during long-term salinity adaptation. Moreover, the osmolality of brackish water (6–18‰) is closer to that of the body of *E*. *sinensis*, thus reduced energy consumption during osmoregulation would contribute to the accumulation of nutrients and/or energy [9]. The percentages of EPA, DHA, and ∑LC-PUFA in ovarian neutral lipids and polar lipids decreased with increasing salinity. This might be because increased LC-PUFA, such as EPA and DHA, could improve membrane fluidity and permeability, which would help maintain the intracellular and extracellular osmotic and ionic balance [15]. Previous studies have demonstrated that elevating salinity could promote ovarian development and maturation of female *E*. *sinensis* [10]. In addition, our research found that the contents of EPA, DHA, and ∑LC-PUFA in the ovaries of female *E*. *sinensis* decreased during ovarian development and maturation (unpublished data). Therefore, the decrease of EPA, DHA, and ∑LC-PUFA levels in the ovaries of female *E*. *sinensis* might also be related to the enhanced ovarian development and maturation caused by brackish water; however, the underlying mechanism remains to be determined.

The hepatopancreas is the major organ of lipid storage and metabolism in crustaceans, and its fatty acid composition can reflect the adaptation of crustaceans to salinity to a certain extent [14, 22]. In this study, the results of PCA showed that the fatty acid composition of the hepatopancreas of male crabs in the 0‰ treatment group was significantly different from that in the 12‰ and 18‰ treatment groups, which indicated that brackish water could affect the fatty acid composition of the hepatopancreas of male crabs. The percentages of ∑MUFA in neutral lipids in the hepatopancreas of male crabs increased significantly with increasing salinity. This might be because of the osmolality of brackish water is closer to that of the body of male *E*. *sinensis*; therefore, reduced energy consumption is required for osmoregulation [4, 9], resulting in more energetic lipids accumulating in the hepatopancreas. The percentages of EPA, DHA, ∑n-3PUFA, and ∑LC-PUFA in neutral lipids and polar lipids in the hepatopancreas of males showed an overall decreasing trend with increasing salinity, which is consistent with the results observed in the mud crab *Scylla serrata* [22]. For female crabs, the results of PCA analysis showed that the fatty acid profile in their hepatopancreas in the 0‰ treatment group was similar to that in the 6‰ and 18‰ treatment groups; however, there was a significant difference between the 0‰ and 12‰ treatment groups. The highest percentages of ∑SFA and ∑MUFA in the neutral lipids and polar lipids of the hepatopancreas female crabs were detected in the 0‰ treatment group, which is in contrast to the results for the male crabs. This may be because the gonadal development of female crabs is generally more dependent on hepatopancreatic lipids than that of males [29, 30]). Additionally, elevated salinity could promote ovarian development of female *E*. *sinensis* [10]; hence the SFA and MUFA in the hepatopancreas might be transported to the ovaries for storage, providing energy for late embryo development, which is consistent with the increased ∑MUFA percentage in the ovaries of the 18‰ treatment group.

In this study, a significantly higher percentage of ∑SFA in neutral lipids of male crabs muscle was detected in the 0‰ treatment group compared with that in the 12‰ and 18‰ treatment groups. Such a result could be explained by the fact that the increasing salinity enhanced the activity frequency of *E*. *sinensis* [10, 43]. The muscle is an important motor organ, and excessive activity frequency might decrease the percentage of SFA in muscle neutral lipids (energetic lipids). However, there was no significant difference in the percentages of ∑SFA, ∑MUFA, and ∑PUFA in the polar lipids of male muscles among all salinity treatments, which is consistent with observation of *E*. *sinensis* during short-term salinity adaptation [24]. For the female crabs, the percentages of EPA, DHA, ∑n-3PUFA, and ∑LC-PUFA in polar lipids were highest in the 0‰ treatment group, which was consistent with a previous study on *S*. *serrata* [22]. This might be because the osmolality difference between the body of female crabs and the ambient solution is higher in the high salinity treatment [4, 10]. Therefore, it is necessary to improve membrane permeability to enhance the absorption of ions and maintain the intracellular ionic balance. It is worth noting that the EPA and DHA in polar lipids are important fatty acids that regulate and maintain membrane integrity and permeability [15]; hence the increasing EPA and DHA levels might be beneficial to the intracellular and extracellular osmotic and ionic balance.

## Conclusion

This study showed that increasing the ambient salinity could promote the accumulation of total lipids and neutral lipids in the hepatopancreas, as well as the polar lipids in the muscles of *E*. *sinensis* after 40 days of salinity adaptation. The fatty acid profiles in the gonads and muscles of adult *E*. *sinensis* were relatively conserved, indicating that they are not susceptible to fluctuating salinity; however, the fatty acid profile in the hepatopancreas was markedly affected by changes in salinity.
